# Plant-Based Sunscreen Emulgel: UV Boosting Effect of Bilberry and Green Tea NaDES Extracts

**DOI:** 10.3390/gels10120825

**Published:** 2024-12-13

**Authors:** Milica Martinović, Ivana Nešić, Dragica Bojović, Ana Žugić, Slavica Blagojević, Stevan Blagojević, Vanja M. Tadić

**Affiliations:** 1 Department of Pharmacy, Faculty of Medicine, University of Niš, Boulevard Dr. Zorana Djindjića 81, 18108 Niš, Serbia; milica.martinovic@medfak.ni.ac.rs (M.M.); ivana.nesic@medfak.ni.ac.rs (I.N.); 2 Faculty for Food Technology, Food Safety and Ecology, University of Donja Gorica, Oktoih 1, 20000 Podgorica, Montenegro; dragica.bojovic@galenikacg.me; 3 Department for Pharmaceutical Research and Development, Institute for Medicinal Plant Research “Dr. Josif Pančić”, Tadeuša Koscuška 1, 11000 Belgrade, Serbia; azugic@mocbilja.rs; 4 Department of Physical Chemistry and Instrumental Methods, Faculty of Pharmacy, University of Belgrade, Vojvode Stepe 450, 11221 Belgrade, Serbia; slavica.blagojevic@pharmacy.bg.ac.rs; 5 The Institute of General and Physical Chemistry, Studentski Trg 12/V, 11158 Beograd, Serbia; sblagojevic@iofh.bg.ac.rs

**Keywords:** natural deep eutectic solvents, NaDES, SPF, sun protection, UV booster, antioxidants, texture analysis, gels, emulgels, creams, emulsions

## Abstract

Natural deep eutectic solvents (NaDES) were employed for the extraction of bilberry and green tea leaves. This study explored the incorporation of these NaDES extracts into various carrier systems: hydrogels, emulsions, and emulgels stabilized with hydroxyethyl cellulose or xanthan gum. The results demonstrated that, when combined with synthetic UV filters, the NaDES extracts significantly enhanced the SPF and improved the antioxidant properties of the formulation. Although NaDES extracts cannot fully replace synthetic UV filters (homosalate, ethylhexyl methoxycinnamate, and benzophenone-4), they can serve as effective UV boosters, significantly enhancing the SPFs of formulations containing UV filters. Hence, the SPF of the formulation could be improved without increasing the concentrations of synthetic filters. Moreover, NaDES extracts, unlike UV filters, significantly increased the antioxidant potential of the formulations. Among the carriers, hydrogels with xanthan gum and emulgels with hydroxyethyl cellulose achieved the highest SPFs when containing both NaDES extracts and synthetic filters. A texture analysis further revealed that the NaDES extracts positively impacted the mechanical properties of the formulations by increasing their cohesiveness, thus enhancing their physical stability under mechanical pressure. These findings pave the way for further research into NaDES-based formulations, including in vivo testing, to optimize and confirm their efficacy on human skin and validate NaDES extracts as eco-friendly ingredients in cosmetics, with antioxidant and UV boosting potential.

## 1. Introduction

It is well known that uncontrolled sun exposure can lead to both acute skin reactions, such as burns and allergies, and chronic effects, including esthetic concerns like photoaging or more serious conditions like photocarcinogenesis. Therefore, the daily use of sunscreens with UV filters is recommended. These substances are cosmetically active ingredients that either absorb or reflect UV radiation, providing skin protection. However, growing concerns over synthetic UV filters (linked to allergic reactions, hormonal disruptions, and environmental harm) have sparked increased public and scientific interest in finding plant-based, natural alternatives. Consequently, plant isolates (such as extracts, oils, and plant-derived compounds) are being explored as potential photoprotective agents [1,2].

Plant isolates represent a rich source of secondary metabolites that possess numerous biological properties and may be used as substitutes for synthetic molecules in pharmaceutical or cosmetic products. As natural and environmentally friendly alternatives, their popularity and relevance in the research field continue to grow, and they are being increasingly used in the formulation of various preparations [3]. Given that it is inherently difficult to achieve a high degree of UV protection using plant extracts per se, some studies have explored the potential application of plant isolates as so-called UV boosters, i.e., substances that enhance the activity of synthetic UV filters. In this way, the spectrum of protection provided by synthetic UV filters can be expanded and their efficacy preserved (or even improved), while allowing the use of lower concentrations of synthetic UV filters, resulting in an improved safety profile for the entire formulation. Unlike synthetic UV filters, where higher concentrations increase the risk of adverse effects (potential endocrine disruptors, allergenic substances, etc.), in the case of plant extracts, not only does increasing their concentration reduce the likelihood of adverse effects, but it also enhances the potential for other beneficial effects on the skin that certain plant extracts may have. Moreover, synthetic UV filters pose threats to the ecological environment, while plant extracts are biodegradable and environmentally friendly, if green extraction solvents are used for their preparation [4,5,6,7].

To achieve the best effect from plant isolates, it is necessary to choose the optimal extraction method and the appropriate extraction solvent [8]. Due to increased environmental awareness and the tendency to produce extracts with an improved safety profile, green solvents, as biodegradable substances of natural origin, are emerging as promising candidates in the field of extraction agents. Examples of such extraction solvents are natural deep eutectic solvents (NaDES), which are formed by combining hydrogen bond donors and hydrogen bond acceptors of natural origin. Upon mixing, they have a lower melting point than the individual components, resulting in liquid NaDES [9,10].

In this context, our previous investigation revealed extracts of green tea leaves (*Camelliae sinensis non fermentatum folium*, *Camellia sinensis* (L.) Kuntze, Theaceae) and bilberry leaves/fruit (*Myrtilli folium*, *Myrtilli fructus*, *Vaccinium myrtillus* L., Ericaceae) prepared with NaDES to possess higher phenolic content and better antioxidant activity in comparison to conventional solvents (water and ethanol) [11]. Based on the results, a bilberry leaf extract (malic acid + glycerol; BLMG) and a green tea leaf extract (tartaric acid + sorbitol; GLTS) were selected and further used in this study. First, their UV-protective activity was examined, after which they were incorporated into different carriers, followed by the evaluation of the organoleptic properties, sun protection factor (SPF) values, antioxidant activity, and textural characteristics of the developed formulations.

It is commonly accepted that carriers serve as systems designed to protect active substances, reduce their degradation, and improve their efficacy and bioavailability at the target site of action. Topical delivery systems are used for the delivery of biological and cosmetic active substances intended to achieve localized effects on the skin, with emulsions being the most commonly used systems for this purpose. Emulsions may serve as carriers for both hydrophilic and lipophilic substances, and they offer highly favorable physicochemical properties, spreadability, and biocompatibility with the structure of the skin. On the other hand, hydrogels are very promising drug delivery vehicles, composed of a high percentage of water and a polymer responsible for forming a 3D network that may capture the active substance. However, their main drawback is the inability to incorporate hydrophobic substances, which may be overcome by the development of emulgels—systems that combine the properties of both emulsions and gels [12,13,14].

Although all stated types of topical vehicles are popular and well accepted by users, they possess very different physicochemical characteristics; therefore, for a certain active substance, it is necessary to compare them and determine which one is the most suitable carrier for the intended preparation purpose. Therefore, the aim of this study was to prepare hydrogels (with xanthan gum (XG) and hydroxyethyl cellulose (HEC)), emulsions (stabilized with Olivem 1000), and emulgels (as a combination of a hydrogel and emulsion) and to evaluate them as carriers of NaDES extracts (BLMG and GLTS) alone or in combination with synthetic UV filters (mixture of homosalate, ethylhexyl methoxycinnamate, and benzophenone-4). The objective was to conduct an assessment of the extracts’ impacts on the degree of UV protection of the formulated products, as well as the influence of the carriers on the UV-protective and antioxidant activity of the preparations themselves. In addition, the aim was also to establish whether the NaDES extracts could replace synthetic UV filters and to what extent, as well as how the choice of carrier affects this.

## 2. Results and Discussion

**Figure 1:**
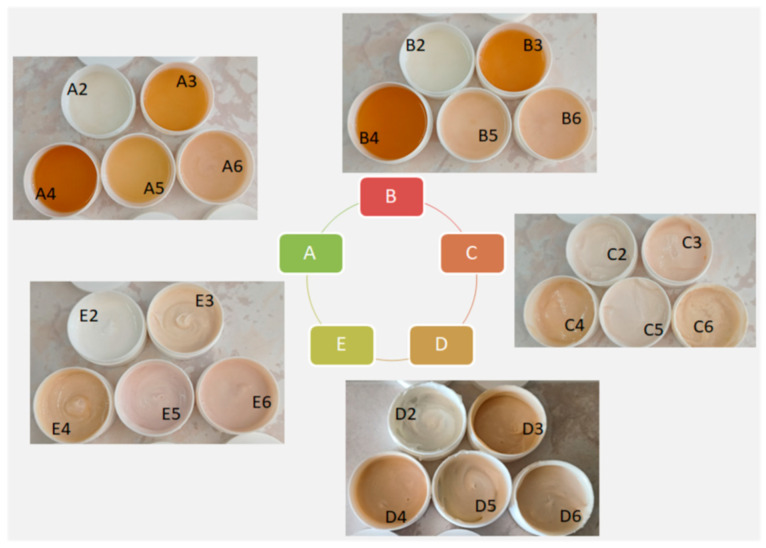
Prepared semi-solid samples (A—hydrogels with XG, B—hydrogels with HEC, C—emulsions with Olivem 1000, D—emulgels with XG, E—emulgels with HEC).

For the purpose of this study, 30 semi-solid samples were prepared. After the preparation of the samples (Figure 1), their stability was assessed and their physicochemical and organoleptic characteristics were noted.

The pH values of all samples were adjusted to 4.5. All samples preserved their organoleptic characteristics and pH after the centrifuge assay, accelerated stability assay, and long-term stability assay. Neither phase separation nor creaming was detected. All samples were homogenous and glossy.

The color of the samples was influenced by the concentrations of the NaDES extracts. The samples with higher concentrations of NaDES extracts had darker tones (Figure 1). The addition of UV filters also influenced the color in the samples marked as 5 and 6, where both NaDES extracts and UV filters were present.

The placebo samples (marked as 1) and the emulsion and emulgel samples with UV filters (C2, D2, E2) had no odor, while the gel samples (A2, B2, A5, B5, A6, B6), where the UV filters were primarily dissolved in ethanol, had an ethanol odor. Other samples containing NaDES extracts had a pleasant smell.

**Figure 2:**
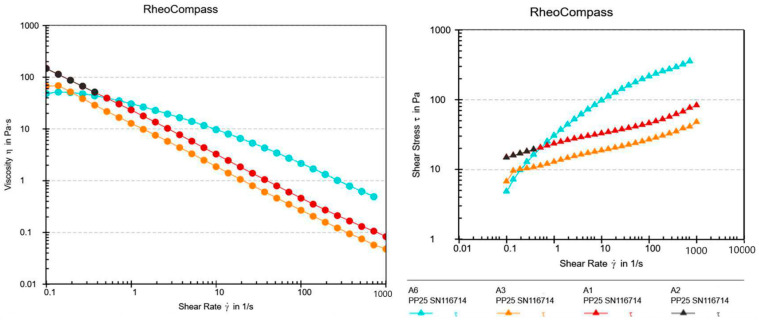
Viscosity and shear stress as a function of the shear rate for the hydrogels with XG (A1, A2, A3, and A6).

**Figure 3:**
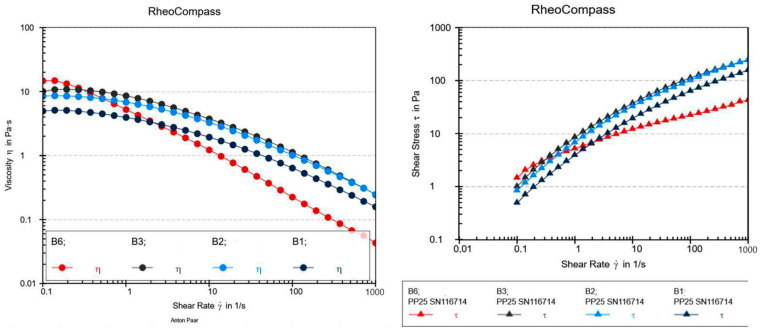
Viscosity and shear stress as a function of the shear rate for the hydrogels with HEC (B1, B2, B3, and B6).

Since two different gelling agents were used in different concentrations, a rheological analysis of XG gels A1, A2, A3, and A6 and HEC gels B1, B2, B3, and B6 was conducted to compare the viscosity of the gels and the influence of the addition of synthetic UV filters, NaDES, and a combination of synthetic UV filters and NaDES on their viscosity, as well to observe their flow curves (Figure 2 and Figure 3).

All analyzed gel samples exhibited non-Newtonian pseudoplastic behavior (a decrease in viscosity with an increase in the shear rate), which indicated their good spreadability. Pseudoplasticity (shear-thinning behavior) is essential for gels because it ensures high stability when at rest, thanks to elevated viscosity at low shear rates (in storage or at the application site), while also enabling easy spreadability under force, as the viscosity decreases at higher shear rates (during application) [15]. Gels with XG demonstrated greater resistance to shear compared to gels with HEC. This indicated a stronger three-dimensional network formed by XG compared to HEC, resulting in their better stability. It was observed that the presence of both NaDES extracts and UV filters resulted in slight changes in the flow curve in both gels with different gelling agents (A6 and B6), which was also observed in the study by Calixto et al. [16], probably as a result of the interaction between the UV filters and NaDES extracts.

### 2.1. Determination of UV-Protective Activity

As UV filters are synthetic substances, the use of plant extracts and naturally derived substances for sun protection represents a new trend in the cosmetics industry. In recent years, there has been a significant increase in the use of plant extracts due to the growing consumer interest in “green” and “natural” ingredients in the final products. Numerous studies published in the scientific literature highlight the photoprotective activity of natural compounds/products, attributed to their ability to absorb UV rays, their antioxidant action, and their DNA-protective properties. The complex composition of plant extracts is responsible for the wide range of biological properties that they can provide. Additionally, their safety and the fact that almost all synthetic UV filters have allergenic potential or may cause adverse effects in humans or the environment further stimulate interest in plant extracts and naturally derived substances [2,17,18,19].

Various methods for SPF testing have been developed. The officially accepted methods for the measurement of UV-protective properties are in vivo studies, while in vitro methods are still in the process of harmonization; currently, no in vitro method has been globally accepted as an equivalent to the mentioned “gold standard” [20]. Regardless of the officially accepted methods, in vitro methods are most commonly used due to their simplicity, speed of execution, and cost-effectiveness. They are especially useful in the phase of product development. Moreover, in vitro methods are prioritized as testing methods since in vivo methods raise ethical and safety questions [21,22]. All in vitro methods applied to determine the level of sun protection can be divided into two groups: methods where the determination of the UV absorption capacity is performed using dilute solutions (dilution methods) and methods where the absorption or transmission of UV radiation is measured through the thin film of a tested preparation applied to an appropriate UV-transparent substrate (3 M Transpore tape, biomembranes, polymethyl methacrylate (PMMA) plates, etc.) [20,23].

In our study, we employed the dilution method for the SPF determination of the NaDES extracts and the latter for the SPF determination of the developed semi-solid samples. The SPF of the crude NaDES extract BLMG was 8.12, while the SPF of the NaDES extract GLTS was 4.20. Dilution methods are regarded as less precise since they rely on the absorbance of the solution, while sunscreens in real-life conditions are not used in a dissolved form [20]. The other method that was used in this study was the standardized ISO 24443:2021 method, where the SPF was determined through thin layers of the samples, as this method is considered more accurate [24]. In order to check the accuracy of both methods, we compared the results obtained with them (Table 1).

In the current study, all placebo samples with no UV filters or NaDES extracts, marked as 1 (A1, B1, C1, D1, and E1), showed SPFs ≈ 0, which means that no tested carrier provided sun protection. The measured SPFs for samples with 12% of UV filters, marked as 2 (A2, B2, C2, D2, and E2), ranged from 8.3 (D2) to 14.3 in the case of the emulgel with HEC (E2). Prior to the in vitro study, in silico calculation was performed for the prediction of the SPF values of the preparations containing 4% of homosalate, 4% of benzophenone-4, and 4% of ethylhexyl methoxycinnamate, as well as those containing 2% of homosalate, 2% of benzophenone-4, and 2% of ethylhexyl methoxycinnamate. According to a freely available in silico web tool, namely the BASF Personal Care Sunscreen Simulator, the former’s SPF was predicted to be 12.3, while the latter’s was 7.2. The predicted UVA-PF was 2.3 and 1.8, respectively.

The initial intention was to fully replace the synthetic UV filters in the formulations; therefore, the samples marked with “3” contained the NaDES extracts in the same concentrations as the UV filters incorporated into the samples marked as “2”. However, the samples with 12% of the NaDES extracts and no UV filters (A3, B3, C3, D3, and E3) showed SPFs ranging from 0.18 to 0.78 (Table 1), indicating that very low sun protection could be achieved by incorporating these concentrations of the extracts in the formulation. On the other hand, when increasing the concentrations of the NaDES extracts twice, the SPF values of samples A4, B4, D4, and E4 more than doubled and were within the range of 0.76–1.22 (Table 1). This result was somehow expected, since the NaDES extracts were used in concentrations of 12% and 24% and the SPF values of the crude NaDES extracts were 8.12 (for BLMG) and 4.20 (for 4.20).

Although the results showed that the prepared NaDES extracts could not be used as complete replacements for UV filters, the calculated SPF values of the samples containing 6% of UV filters and 6% of the NaDES extracts (A5, B5, C5, D5, and E5) and the samples containing 12% of UV filters and 12% of the NaDES extracts (A6, B6, C6, D6, and E6) showed that the NaDES extracts had great potential as UV boosters and that they could increase the SPF value of the final formulation without the need for the further addition of synthetic UV filters (Table 1). For instance, in the case of the emulgel with XG, the SPF of sample D2 containing 12% of UV filters was 8.3. When half of the UV filters were replaced with NaDES extracts (in the case of sample D5), the obtained SPF was 7.3. In the same series of samples, both sample D3 (12% of NaDES) and sample D4 (24% of NaDES) showed SPFs < 1, indicating that the NaDES extracts alone could not enable sufficient UV protection. However, 6% of the NaDES extract in sample D5 almost replaced 6% of the UV filters, leading to an SPF that was just one unit lower than the SPF of the formulation with 12% of UV filters.

The addition of the NaDES extracts increased the SPFs of all samples by at least 1.5. In the case of the hydrogels with XG, the addition of the NaDES extracts (12%) to the formulation with 12% of UV filters led to an increase in the SPF from 11.2 (A2) to 17.2 (A6). This sample was also the one with highest measured SPF in this study.

In the series of hydrogel extracts with HEC, 12% of the NaDES extracts provided SPF 0.41 (B2), while the addition of the same concentration of the extracts increased the SPF of the formulation by 1.3 (SPF(B6)–SPF(B2)) (Table 1).

With regard to the emulsion samples, the difference was even larger. The sole incorporation of NaDES extracts (C2) provided an even lower SPF of 0.18, while their addition in the same concentration increased the SPF by 2.9 (SPF(C6)–SPF(C2)) (Table 1).

More papers are focusing on the hypothesis that various plant extracts could, in part, replace synthetic UV filters, enabling their use in lower concentrations without compromising the target SPF. However, to our knowledge, there are few papers that have focused on examining the potential UV protection effects of NaDES extracts. A spray gel was formulated with Gedong mango and mulberry leaf extracts obtained using NaDES consisting of sodium acetate and lactic acid [25]. Mattioli et al. evaluated glycerol-containing NaDES (proline + glycerol) as potential photoprotective agents able to inhibit effects on the photochemical isomerization of resveratrol [26]. Nevertheless, studies have shown that NaDES extracts can have good antioxidant activity, which indicates their potential for incorporation into sunscreens [27,28].

**Figure 4:**
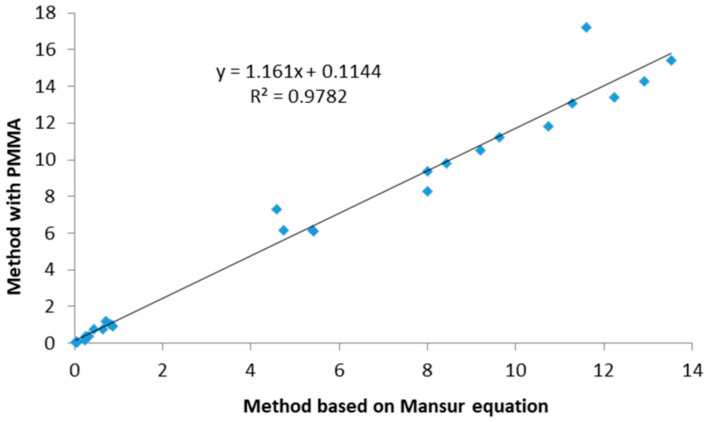
The correlation between the obtained SPF results of the two methods.

The results showed a very strong positive correlation between both methods applied for SPF calculation (Figure 4), which was statistically significant (Pearson correlation coefficient r = 0.98, *p* < 0.01).

**Figure 5:**
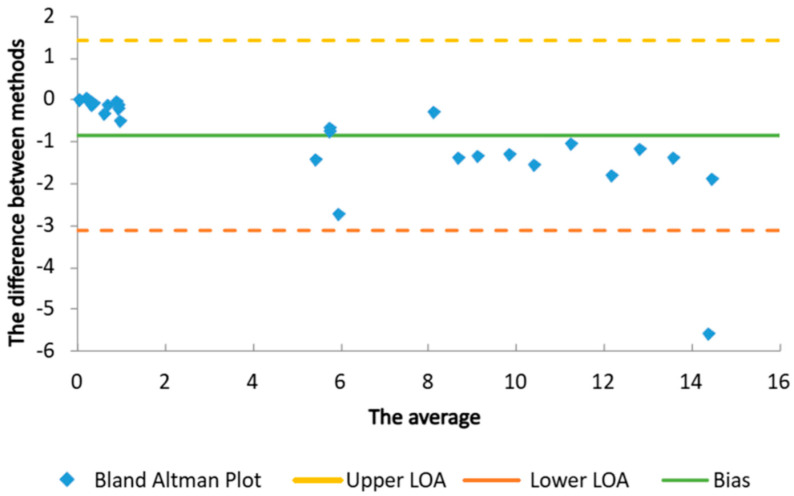
The Bland–Altman plot of the applied in vitro methods for SPF measurement (method based on Mansur equation and method with PMMA plates).

The Bland–Altman plot, as a statistical means for the comparison of two methods, showed a small bias between the results (Figure 5). Besides basic methodological principles, one of the reasons for this bias might be the fact that the methods were performed in different laboratories. The mean difference (bias) between the method based on the Mansur equation and the method with PMMA plates was −0.87, indicating that the first method tends to give slightly lower results compared to the second. The 95% limits of agreement were −3.14 to 1.40, meaning that 95% of the differences between the methods fell within this range. In practice, this suggests that the method based on the Mansur equation may underestimate the SPF values by up to 3.14 units or overestimate them by up to 1.40 units compared to the method with PMMA plates. This bias was statistically significant (*p* < 0.01), showing a systematic difference between the methods. However, due to the small bias and the limits of agreement (Figure 5), these methods can likely be used together or substituted for one another in most cases, with minor adjustments for the bias [29].

Besides the SPF, some other parameters should be considered when characterizing sunscreens. The UVA-PF shows the degree of protection against UVA, and, according to the European Regulation 1223/2009, it should be at least one-third of the SPF [30]. In our study, all samples, except for the placebo samples (A1, B1, C1, D1, and E1) and the samples with 12% of the NaDES extracts (A3, B3, C3, D3, E3), showed the desired UVA/UVB ratio (Table 1). This was even the case for samples with 24% of NaDES extracts and no synthetic UV filters (A4, B4, C4, D4, E4). The highest UVA-PF was measured for sample A6 from the series of hydrogels with XG (UVA-PF = 5.7) and sample E6 from the series of emulgels with HEC (UVA-PF = 5.5). Even regarding protection from UVA rays, it was noticed that it could be significantly improved by the NaDES extracts, indicating that NaDES extracts could also “boost” the UVA-PF. The greatest influence was observed in the case of the hydrogels with XG, where the UVA-PF of sample A3 was lower than 0.1, but, when the same concentration of the extract was added to the sample with synthetic UV filters, the UVA-PF was raised from 3.8 to 5.7 (Table 1).

The critical wavelength (λ) corresponds to the wavelength at which the area under the absorbance curve (AUC) represents 90% of the total AUC in the UV spectrum from 290 to 400 nm. This parameter should be higher than 370 nm, indicating “broad-spectrum” protection [24,31], which was the case with all tested samples in our study (Table 1).

### 2.2. Determination of Antioxidant Activity

Sunscreens should also be able to provide antioxidant effects, given that UV radiation partially leads to tissue damage through the creation of free radicals. In addition to UV protection, the antioxidant effect is another important photoprotective mechanism attributed to plant extracts, as it is known that UV radiation leads to the production of reactive oxygen species. This mechanism of action can be attributed to phenols, flavonoids, anthocyanins, phytoalexins, hydrolyzed tannins, isoflavones, inositol, fatty acids, cyanidin 3-glucoside, and other metabolites. Plant extracts, as powerful antioxidants, have shown potential to protect the skin from UV-induced damage through several mechanisms, including the reduction of oxidative stress, inflammation, immunosuppression, and DNA damage and the suppression of signaling pathways that lead to the photoaging of the skin [32,33].

In our previous work, NaDES extracts demonstrated very good antioxidant potential. The results of DPPH tests revealed that the IC_50_ of BLMG was 0.59 mg/mL, while the IC_50_ of GLTS was 0.25 mg/mL. Their FRAP values were 0.94 mmol Fe^2+^/g DW and 1.66 mmol Fe^2+^/g DW, respectively [11]. While GLTS showed better antioxidant potential, the above results indicate that BLMG offered better UV protection and provided a higher SPF.

**Figure 6:**
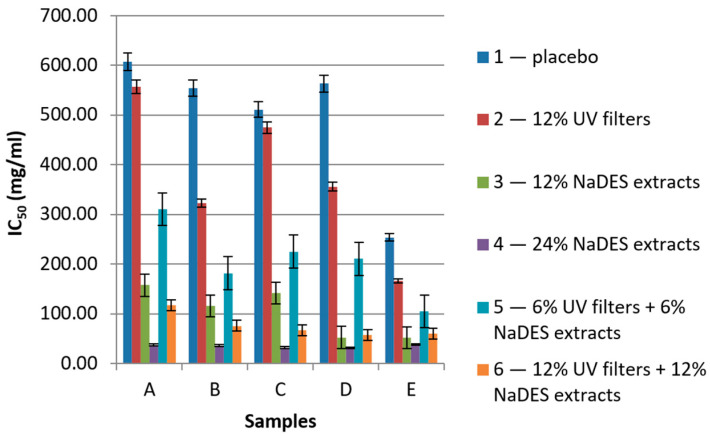
The results of the DPPH assay presented as IC_50_ values (mg/mL) of samples (A—hydrogels with XG, B—hydrogels with HEC, C—emulsions with Olivem 1000, D—emulgels with XG, E—emulgels with HEC).

**Figure 7:**
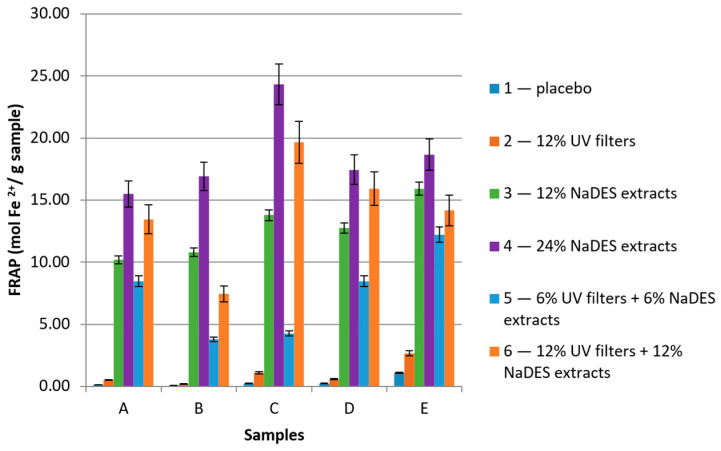
The results of the FRAP assay presented as mol Fe^2+^/g for samples (A—hydrogels with XG, B—hydrogels with HEC, C—emulsions with Olivem 1000, D—emulgels with XG, E—emulgels with HEC).

In the current study, the antioxidant activity of all samples was determined, and the results are presented in Figure 6 and Figure 7.

The antioxidant assays revealed that increasing the concentration of the NaDES extract in the sample led to a significant increase in its antioxidant activity. The best antioxidant activity was observed in the case of samples with 24% of NaDES extracts (A4, B4, C4, D4, and E4), whose DPPH IC_50_ values ranged from 31.09 to 38.61 mg/mL (Figure 6), and the FRAP values ranged from 15.50 to 24.32 mol Fe^2+^/g of sample (Figure 7). On the other hand, the samples with only UV filters (A2, B2, C2, D2, and E2) showed low antioxidant activity that did not statistically significantly differ from the antioxidant activity of the placebo samples, leading to the conclusion that synthetic UV filters per se offer very low protection from free radicals. In the case of combining the UV filters and NaDES extracts, interesting results could be seen. A comparison of the values for samples in series “3” and “6”, as well as samples in series “2” and “6”, led to the conclusion that the presence of UV filters did not notably increase the radical scavenging activity. Nevertheless, the improved antioxidant activity of the samples in series “6” was a result of the NaDES extracts. When comparing series “5” and “6”, it could be noted that, even though the concentrations of the NaDES extracts and UV filters were doubled, the antioxidant activity in most cases was more than doubled. For instance, the IC_50_ value of sample D5 (hydrogel with XG), with 6% NaDES extracts and 6% UV filters, was 210.57 mg/mL. On the other hand, the IC_50_ value of sample D6 (hydrogel with XG), with 12% NaDES extracts and 12% UV filters, was almost four times lower—57.65 mg/mL.

**Figure 8:**
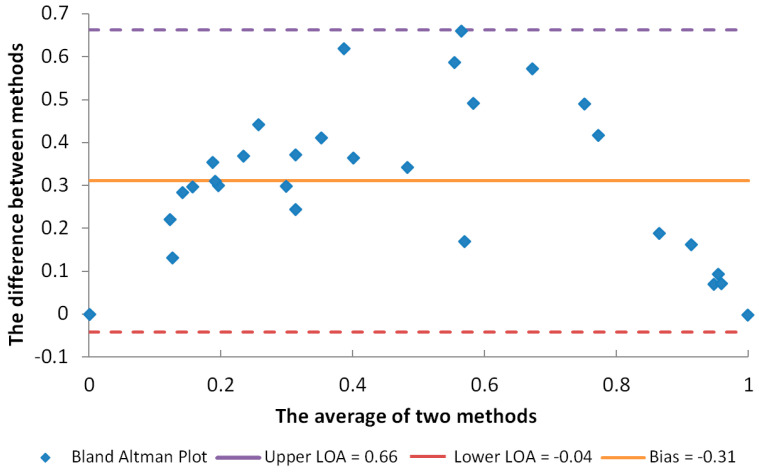
The Bland–Altman plot of the applied antioxidant assays (DPPH and FRAP).

For a comparison of the results of both applied methods, a correlation analysis was conducted and a Bland–Altman plot was created. Since higher FRAP values indicate stronger antioxidant activity and lower DPPH IC_50_ values show better radical scavenging activity, in order to perform the statistical analysis, the results of the DPPH and FRAP assays were normalized and converted into percentages, so that the highest antioxidant activity was marked as 100% and the lowest as 0%. The correlation analysis manifested a strong, statistically significant correlation, with a Pearson correlation coefficient r = 0.84 (*p* < 0.01). The Bland–Altman plot (Figure 8) also showed that all results were within the limit of agreement (LoA). However, the LoA was wide, which indicated that one method could not directly predict the results of the other. One of the reasons for this is that the DPPH assay is based on calculating the radical scavenging activity [22] while the FRAP assay focuses on electron transfer [23], so both assays estimate the antioxidant activity from the perspective of different mechanisms, and they should be used in combination.

### 2.3. Texture Analysis

A texture analysis was used for the further characterization of the developed semi-solid samples. It was of special interest to evaluate the influence of the added UV filters and NaDES extracts on the mechanical characteristics of the tested samples. Although texture analysis was initially developed to assess the mechanical properties of food, in recent years, it has been widely used in the cosmetic industry, also aiming to assess the mechanical characteristics, which are important in terms of both preparation stability and consumer acceptability. Texture analysis is usually conducted using probes, which are immersed in the cosmetic product, mimicking human fingers. During this process, a load vs. time graph is constructed, and some parameters, like the hardness, adhesiveness, and cohesiveness, are measured [34,35]. According to Szczesniak, who gave one of the first classifications of textural characteristics, hardness, as “a force necessary to attain given deformation”, could be described with the terms “soft”, “firm”, or “hard”, while adhesiveness, defined as the work necessary to overcome attractive forces between the surface of the product and other surfaces with which the product comes into contact, can be described with “sticky”, “tacky”, and “gooey”. Elasticity is a parameter indicating the degree at which the deformed material returns to its non-deformed state after removing the deformation force, while cohesiveness is a parameter that denotes the strength of the internal bonds in the tested product [36].

**Figure 9:**
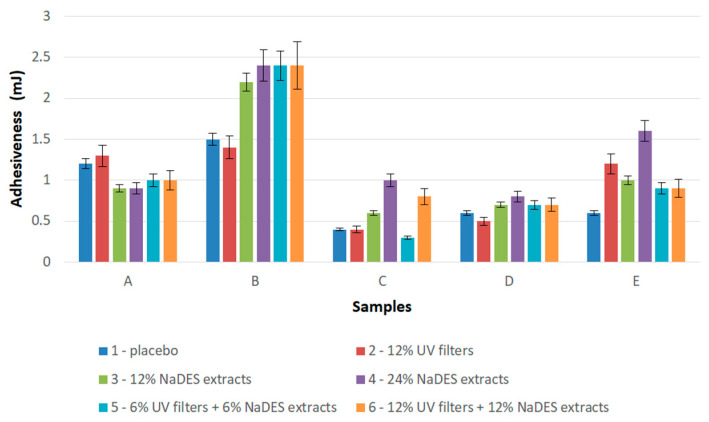
Adhesiveness of semi-solid samples (A—hydrogels with XG, B—hydrogels with HEC, C—emulsions with Olivem 1000, D—emulgels with XG, E—emulgels with HEC).

Adhesiveness is a measure of the stickiness of the preparation; higher stickiness may indicate longer contact between the preparation and the skin [37]. Figure 9 shows that the hydrogel samples (samples A and B) had higher adhesiveness compared to the other samples. The HEC-based hydrogel samples with NaDES extracts (B3–6) had higher adhesiveness compared to the matching XG-based hydrogels (A3–6). The same was found for the emulgel samples—the HEC-based emulgels with NaDES extracts (E3–6) had higher adhesiveness compared to the XG-based emulgels (D3–6). The presence of the NaDES extracts significantly increased the adhesiveness of the preparation. Moreover, in the case of emulsions (samples C3 and C4) and HEC-based emulgels (samples E3 and E4), the adhesiveness increased with the increase in the concentration of NaDES extracts.

**Figure 10:**
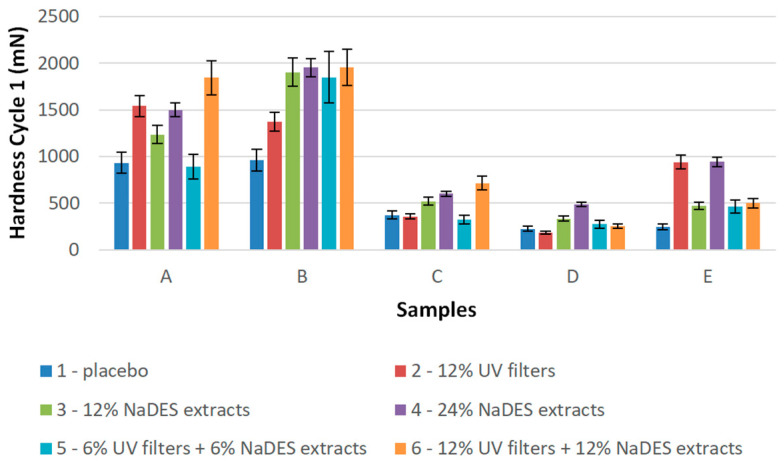
Hardness cycle 1 of the semi-solid samples (A—hydrogels with XG, B—hydrogels with HEC, C—emulsions with Olivem 1000, D—emulgels with XG, E—emulgels with HEC).

It is commonly accepted that the spreadability of a sample is inversely related to its hardness [37,38]. As seen in Figure 10, hydrogels (samples A and B) revealed the highest hardness values and therefore may be considered as the samples that are the most difficult to spread. On the other hand, in terms of spreadability, the emulsions (samples C) and emulgels (samples D and E) showed advantageous properties (Figure 10). The observed behavior of the hydrogels might be connected to their previously discussed higher adhesiveness, indicating that, when the product is stickier, it is also more difficult to spread. In addition, it might be speculated that the presence of the oil phase in emulsions and emulgels may be responsible for reducing the friction and enabling the interaction with the skin lipids and easier rubbing [39].

The standard protocol of texture profile analysis consists of two cycles of probe immersion in the sample, and the hardness was measured after each of these [35,38,40]. The measured hardness values in cycle 2 only slightly differed from the measured hardness values in cycle 1, indicating that the firmness of the samples was preserved despite the pressure of the probe.

**Figure 11:**
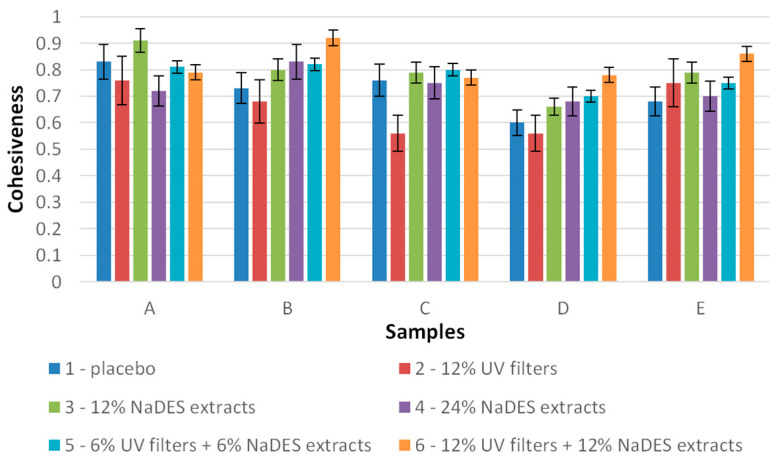
Cohesiveness of semi-solid samples (A—hydrogels with XG, B—hydrogels with HEC, C—emulsions with Olivem 1000, D—emulgels with XG, E—emulgels with HEC).

Cohesiveness is calculated by dividing the areas under the curve from both compression cycles on the load vs. time graph, and its values range from 0 to 1 (no units). While 0 indicates that the structure is demolished by the compression cycle, 1 means that the structure is perfectly preserved [37,38]. Figure 11 reveals that all samples exhibited good cohesiveness, mostly higher than 0.6. The best cohesiveness was measured for hydrogels A3 (prepared with XG) and B6 (prepared with HEC), which was around 0.9. Moreover, it could be noted (Figure 11) that the samples comprising solely UV filters (samples 2) tended to have slightly lower cohesiveness, while, on the other hand, the addition of the NaDES extracts (samples A3, B3, B4, C3, D3, D4, E3) affected the cohesiveness positively, meaning that this parameter did not change or even increased compared to the placebo sample.

Overall, the results demonstrated that the implementation of the selected NaDES extracts in the formulations was necessary to ensure good antioxidant activity. However, the presence of synthetic UV filters was mandatory to achieve the desired SPF. Nevertheless, NaDES extracts can show a UV boosting effect and significantly increase the SPF values of formulations with synthetic UV filters, thus enabling an increase in the SPF without incrementing the concentrations of the synthetic UV filters. Moreover, the choice of carrier can influence not only the textural characteristics but the final SPF. In our study, the best SPF was achieved with the combination of NaDES extracts and synthetic UV filters in samples A6 (hydrogel with XG) and E6 (emulgels with HEC). In the case of the antioxidant activity, the best activity was measured in the emulgels with HEC.

Since our study primarily relied on in vitro methods, the plan for our future work is to conduct an in vivo examination on healthy human volunteers to estimate the UV-protective activity of the semi-solid samples, as well as the influence of the formulations on the skin’s biophysical parameters. Secondly, no mineral UV filters were included in this study. The aim of our future studies will be to investigate the potential UV boosting effects of NaDES extracts on formulations with physical UV filters and higher SPF values.

## 3. Conclusions

This study focused on incorporating NaDES extracts into different types of semi-solid carriers—hydrogels with hydroxyethyl cellulose, hydrogels with xanthan gum, emulsions stabilized with Olivem 1000, emulgels with hydroxyethyl cellulose, and emulgels with xanthan gum. The results have shown that incorporating NaDES extracts of bilberry leaves (NaDES: glycerol+malic adic) and green tea leaves (NaDES: sorbitol+tartaric acid) leads to significant improvements in the SPFs of formulations with synthetic UV filters, indicating their UV boosting potential. In the case of the hydrogel with xanthan gum, the addition of NaDES extracts increased the SPF by 6. These results indicate that, since NaDES extracts can enhance significantly the SPFs of synthetic UV filters without increasing the concentrations of synthetic UV filters, they possess potential to replace a certain part of the synthetic UV filters, as natural and safer alternatives. In addition, the NaDES extracts significantly contributed to the antioxidant activity of the obtained formulations. These extracts also contributed to the textural characteristics of the formulations, via increasing their cohesiveness and adhesiveness. The results of this study open the door for additional research into NaDES-based formulations and the establishment of NaDES extracts as safer, biodegradable, and environmentally friendly ingredients in cosmetics.

## 4. Materials and Methods

### 4.1. Materials

The herbal materials used in this study were bilberry leaves (*Myrtilli folium, Myrtilli fructus*, *Vaccinium myrtillus* L., Ericaceae) and green tea leaves (*Camelliae sinensis non fermentatum folium*, *Camellia sinensis* (L.) Kuntze, Theaceae). Their identities were confirmed at the Herbarium of the Faculty of Pharmacy, University of Belgrade (Belgrade, Serbia), where the voucher specimens were deposited (CSNonF_1121 and VML_0921).

For NaDES preparation, tartaric acid and malic acid, purchased from Centrohem (Stara Pazova, Serbia), as well as sorbitol and glycerol, purchased from Comcen (Belgrade, Serbia), were used.

The antioxidant and UV-protective activity of the extracts was assessed using the following analytical-grade reagents purchased from Sigma-Aldrich (St. Louis, MO, USA): acetate buffer, 2,4,6-tripyridyl-s-triazine (TPTZ), 1,1-diphenyl-2-picrylhydrazyl (DPPH), methanol, and ethanol (70%, *v*/*v*).

For the preparation of the hydrogel, emulsion, and emulgel formulations, the following raw materials were used: gelling agents, i.e., xanthan gum and hydroxyethyl cellulose from Chem Point (Krakow, Poland); potassium sorbate from Comcen (Belgrade, Serbia); caprylic/capric triglyceride (Myritol^®^ 318), homosalate, ethylhexyl methoxycinnamate, and benzophenone-4 from BASF (Ludwigshafen, Germany); Olivem 1000^®^ (INCI: sorbitan olivate (and) cetearyl olivate) and Olivem Feel^®^ (INCI: cetearyl alcohol (and) cetyl palmitate (and) sorbitan palmitate (and) sorbitan oleate) from Hallstar (Chicago, IL, USA). Purified water was obtained from the Faculty of Medicine (University of Niš, Niš, Serbia).

### 4.2. NaDES Extract Preparation

NaDES tartaric acid + sorbitol (TS) and malic acid + glycerol (MG) were prepared according to the procedure described in our previous paper, which was based on stirring, heating, and melting the components mixed at a mole ratio of 1:2, until the formation of a colorless transparent liquid [11].

Ultrasound-assisted extraction was performed for 30 min at 50 °C, after which the samples were centrifuged and extracted, and the supernatant was collected and used in further work [11].

### 4.3. SPF Calculation Using Mansur Equation

The determination of the UV absorption capacity was carried out using the dilution method based on the application of the Mansur Equation (1) [41].

(1) $$SPF=CF\times \sum_{290}^{320}EE\left(\lambda \right)\times I\left(\lambda \right)\times Abs(\lambda )$$

EE(λ) indicates the erythemal effect of solar radiation at a specific wavelength, I(λ) refers to the intensity of sunlight at a certain wavelength, and Abs(λ) is the absorbance measured by the spectrophotometer at a given wavelength. CF represents the correction factor. The values of EE(λ) × I(λ) are constants and were calculated by Sayre et al. [42].

This procedure involved weighing 0.5 g of the extract and transferring it into a standard 50 mL flask, which was then filled with ethanol up to the mark. A series of dilutions with ethanol were performed until a dilution of 0.2 mg/mL was achieved. The absorption spectrum was recorded for the prepared samples, and the absorbance was measured at every 5 nm in the range of 290 to 320 nm using an Evolution 60 spectrophotometer, Thermo-Fisher Scientific (Waltham, MA, USA).

### 4.4. Semi-Solid Samples

Samples from series A and B were hydrogels; samples from series C were creams (emulsions); and those from series D and E were emulgels. The gelling agent used for the preparation of series A and D was xanthan gum (XG), while the gelling agent used for the preparation of series B and E was hydroxyethyl cellulose (HEC). Both gelling agents are hydro-soluble polymers of natural origin that form clear dispersions and are generally used to stabilize products and increase the viscosity of formulations. HEC is derived from cellulose, a natural polymer originating from plants, while XG is a fermentation product of *Xanthomonas campestris* bacteria [43]. XG forms hydrogels at very low concentrations, while HEC forms hydrogels at slightly higher concentrations [44]; therefore, XG was incorporated in the hydrogels from series A in 1%, while HEC was used in 2% in the hydrogels from series B. The emulsions (series C) and emulgels (series D and E) were stabilized with Olivem 1000^®^ (INCI: sorbitan olivate (and) cetearyl olivate). This emulsifier is of natural origin, and it is PEG-free and biodegradable (since it consists of a complex mixture of fatty acids similar to skin lipids) [45].

In each series of preparations, there were 6 types of samples. Samples marked as “1” were placebo samples, while samples marked as “2” contained 12% in total of UV filters (4% of homosalate + 4% of ethylhexyl methoxycinnamate + 4% of benzophenone-4). These UV filters were chosen as they are commonly used as cosmetic actives. Ethylhexyl methoxycinnamate offers broad-spectrum protection from both UVB and UVA, benzophenone-4 protects predominantly from UVA, and homosalate protects from UVB and serves as a stabilizer for other UV filters [17]. All of the synthetic UV filters used in this study can be found in the list of UV filters allowed in cosmetic products within Annex VI of EC Cosmetic Regulation 1223/2009, where their maximum allowed concentration is stated (for ethylhexyl methoxycinnamate, benzophenone-4, and homosalate, it is 10%, 5%, and 7.34%, respectively) [46]. These synthetic UV filters are also approved in the USA, with maximum authorized concentrations of 10% for each of them [47].

Samples marked as “3” contained 12% of NaDES extracts (6% of BLMG and 6% of GLTS), while samples marked as “4” contained twice the concentration of NaDES extracts (12% of BLMG and 12% of GLTS). The remaining samples contained a mixture of synthetic UV filters and NaDES extracts. While samples marked as “5” contained the mixture in a total of 12% (6% of NaDES extracts + 6% of synthetic UV filters), samples marked as “6” contained 12% of NaDES extracts and 12% of synthetic UV filters.

#### 4.4.1. Hydrogel Preparation

Hydrogels prepared using XG as a gelling agent were marked A1–A6 (Table 2) and those prepared using HEC were marked B1–B6 (Table 3).

The gelling agent (XG or HEC) was firstly dispersed in a mixture of water, glycerin, and a preservative (potassium sorbate) at room temperature with constant stirring using an RW 16 basic propeller rotary laboratory stirrer (IKA Werke, Staufen, Germany) until a homogenous gel was formed. Since the UV filters (benzophenone-4, homosalate, and ethylhexyl methoxycinnamate) were not soluble in water, they were dissolved in ethanol and the solution was incorporated into the prepared gels A2, A5, A6, B2, B5, and B6. The NaDES extracts were incorporated in the same manner into the prepared gels A3–A6 and B3–B6.

#### 4.4.2. Cream (Emulsion) Preparation

Creams prepared using Olivem 1000^®^ (INCI: sorbitan olivate (and) cetearyl olivate) as an emulsifier were marked C1–C6. Their qualitative and quantitative compositions are presented in Table 4.

The emulsions (C1–C6) were prepared following a standard emulsion preparation protocol [14] based on the separate heating of the water and oil phases, combined with their consequent stirring using an RW 16 basic propeller rotary laboratory stirrer (IKA Werke) until the emulsion reached room temperature. Benzophenone-4 was dissolved in a mixture of homosalate and ethylhexyl methoxycinnamate, and the mixture of UV filters was added to formulations C2, C5, and C6 at a temperature of 40 °C. The NaDES extracts were added to formulations C3–C6 at the same temperature, in order to prevent potential thermodegradation at higher temperatures.

#### 4.4.3. Emulgel Preparation

Emulgels prepared using XG as a gelling agent were marked as D1–D6, and those prepared using HEC were marked as E1–E6. Their qualitative and quantitative compositions are presented in Table 5 and Table 6.

In order to prepare the emulgels, the oil phase and the water phase were separately heated up to 70–80 °C and later mixed until cooled to room temperature using an RW 16 basic propeller rotary laboratory stirrer (IKA Werke, Staufen, Germany). The difference in the preparation of the emulsions and emulgel samples was that the water phase intended for emulgel preparation was gelled and heated prior to mixing with the heated oil phase.

### 4.5. Physicochemical Characterization of the Samples

#### 4.5.1. Physicochemical and Organoleptic Characterization

The prepared samples were analyzed organoleptically. Their color, thickness, appearance, and odor, as well as potential phase separation or creaming, were estimated by visual observation and touch.

The pH values of all samples were measured potentiometrically using a pH meter electrode (pH 211 Microprocessor pH Meter, Hanna Instruments, Woonsocket, RI, USA) and adjusted to 4.5 with the addition of sodium hydroxide.

#### 4.5.2. Stability Assays

The prepared samples were subjected to organoleptic characterization and pH measurement after the centrifuge assay (laboratory centrifuge, at 25 °C and at 3000 rpm for 15 min), after an accelerated stability test (the samples were exposed to three different temperatures for 24 h—room temperature of 21 ± 2 °C, freezer temperature of 5 ± 2 °C, and high temperature of 45 ± 2 °C), and after a long-term stability test (after 1 month of storage at room temperature).

#### 4.5.3. Rheology Analysis

The rheological analysis of the prepared gels was performed with an Anton Paar rheometer (MCR 102 e) at a constant temperature of 25 ± 0.1 °C, using a serrated plate–plate P35/Ti/SE measuring geometry. The gap thickness between the plates was 0.5 mm [48]. Temperature equilibration was carried out for 3 min prior to the beginning of the measurements. The Rheocompass version 1.31 software (Anton Paar, Graz, Austria) was used to control the device and acquire the measured data.

#### 4.5.4. Texture Analysis

The CT3 texture analyzer (Brookfield, AMETEK Inc., Middleborough, MA, USA) was used to perform the texture profile analysis (TPA). The working principle of the device is based on doubly immersing the cone probe (TA-STF) into a sample cup filled with the tested sample, which imitates the movement of a human finger dipped into the cream. The trigger load for the probe was set at 20 mN, and, when it was achieved, the cone test speed was 1 mm/s (the speed of the probe penetrating the sample), and the target value was set at 5 mm (the depth of penetration). The following texture parameters were measured: hardness cycle 1, hardness cycle 2, cohesiveness, and adhesiveness.

### 4.6. UV-Protective Activity of the Semi-Solid Samples

#### 4.6.1. Calculation of SPF of Semi-Sold Samples Using Mansur Equation

The SPF was firstly calculated using the spectrophotometric dilution method based on the application of the Mansur Equation (1) described in Section 4.3. The samples for the assay were prepared with a series of dilutions of each gel/emulsion/emulgel in ethanol until a concentration of 2 mg/mL was reached. Afterwards, the SPF measurements were conducted in the same manner as the SPF measurements of the NaDES extracts.

#### 4.6.2. Calculation of SPF and UVA-PF of Semi-Sold Samples Using PMMA Plates

For the determination of the SPF, the method in which the absorption of UV radiation through the tested preparation applied to an appropriate substrate was also used. The SPF and UVA-PF were determined by applying the method ISO 24443:2021—Cosmetics—Determination of sunscreen UVA photoprotection in vitro [24]. This method is based on measuring the absorbance of the sample on a polymethyl methacrylate (PMMA) plate in the 290 to 400 nm range. The sun protection factor (SPF) is then determined using calculations developed by Diffey and Robson [49]. The PMMA plate manufacturer was Helioscreen (Creil, France), model Helioplate HD 6 (lot 32/2021), with the following transmission values: 290 nm min 60%, 300 nm min 69%, 320 nm min 81%.

The procedure was the following: 50 mg of the sample was spread across the entire surface of the PMMA plate (25 cm^2^), measured on an analytical balance with four decimal places. The surface of the plate was rough, with depths of 4.5 µm. The sample was left to dry for 30 min at room temperature and in the dark. Then, the absorbance was measured between 290 and 400 nm and between 320 and 400 nm to determine the SPF and UVA-PF. The spectrophotometer, M 40—Zeiss, was equipped with an integrating sphere, and each point was measured three times, with the average value calculated. A glycerin-based preparation with a proven SPF of 20 was used as the control sample.

The calculations were performed using Equations (2) and (3), where E is the CIE erythemal spectral efficiency, S is the solar spectral irradiance, and T is the spectral transmission of the sample at individual wavelengths [50].

(2) $$SPF=\frac{\sum_{290}^{400}E\left(\lambda \right)\times S(\lambda )\times d(\lambda )}{\sum_{290}^{400}E\left(\lambda \right)\times S(\lambda )\times T(\lambda )\times d(\lambda )}$$

(3) $$UVA-PF=\frac{\sum_{320}^{400}E\left(\lambda \right)\times S(\lambda )\times d(\lambda )}{\sum_{320}^{400}E\left(\lambda \right)\times S(\lambda )\times T(\lambda )\times d(\lambda )}$$

#### 4.6.3. In Silico SPF Prediction

For the purpose of the in silico SPF prediction of the formulations with synthetic UV filters, the BASF Sunscreen Simulator was used, which is available at the website https://sunscreensimulator.basf.com/ (accessed on 5 September 2024).

### 4.7. Antioxidant Activity of Semi-Solid Samples

#### 4.7.1. DPPH Radical Scavenging Activity

The DPPH radical scavenging activity was estimated by applying a method adapted from Brand-Williams et al. [51]. The procedure is based on mixing 300 µL of the test solution (the semi-solid sample diluted in methanol in 5 different concentrations) and 2.7 mL of freshly prepared methanol DPPH solution (0.04 mg/mL). The absorbance of the mixture was recorded after a 30 min incubation period (room temperature, in the dark) at 517 nm. Methanol was used as a blank, while the control solution (consisting of 2.7 mL of methanol DPPH solution and 300 µL of methanol) was used to calculate the free radical scavenging activity via Equation (4):

DPPH radical scavenging capacity (%) = [(A_C_ − A_S_)/A_C_] × 100 (4)

A_S_ was the absorbance of the test solution treated with the DPPH radical solution;

A_C_ was the absorbance of the control solution.

The results were expressed as IC_50_ values.

#### 4.7.2. Ferric Ion Reducing Antioxidant Power (FRAP Assay)

The antioxidant power of the semi-solid samples was determined with a slightly modified spectrophotometric FRAP assay method from Benzie and Strain [52], conducted on an Evolution 60 UV/Vis spectrophotometer (Thermo Fisher Scientific, Waltham, MA, USA). This spectrophotometric method is based on recording the absorbance at 593 nm of a mixture of 100 µL of different solutions, previously used in the DPPH assay, and 3.0 mL of freshly prepared FRAP reagent. The FRAP reagent was obtained by mixing 25 mL of 300 mM acetate buffer pH 3.6, 2.5 mL of 10 mM TPTZ (2,4,6-tripyridyl-s-triazin) solution in 40 mM HCl, and 2.5 mL of 20 mM FeCl_3_ × 6H_2_O. The FRAP value was calculated from the calibration curve of FeSO_4_ × 7H_2_O standard solutions, covering the concentration range of 100–1000 mmol/L (y = 0.5273x + 0.0771, the linear regression at r^2^ > 0.99) and expressed as mol Fe^2+^/g semi-solid sample.

### 4.8. Statistical Analysis

The results are presented as the average results of three measurements ± standard deviation. To analyze the statistical differences between the groups, a one-way analysis of variance (ANOVA) was used with post hoc analysis, and the differences at *p* < 0.05 were considered statistically significant. Pearson correlation analysis and a Bland–Altman plot were used to compare the methods applied to measure the UV-protective properties of the samples, as well as for both methods applied to measure the antioxidant activity of the tested samples. The statistical analysis was conducted using the IBM SPSS Statistics version 22 software, while all graphs in this paper were constructed using the Microsoft Excel 10 software.

## Figures and Tables

**Table 1:** Calculated SPF and UVA-PF values using dilution method (Mansur equation) and PMMA plates (Robert and Diffey method).

Sample	SPF Mansur Equation	SPF PMMA Plates	UVA-PF (ISO 24443:2021 [24])	UVA/SPF	UVA/UVB	Critical λ (nm)
Reference product	-	20.3 ± 0.5	≈1/3 SPF	max 1	≈0.5	min 370 nm
Samples A—Hydrogels with XG						
A1	0.03 ± 0.01	0.05 ± 0.01	<0.02	-	-	381
A2	9.64 ± 0.2	11.2 ± 0.1	3.8 ± 0.1	0.339	0.51	380
A3	0.25 ± 0.01	0.40 ± 0.01	<0.10	-	-	379
A4	0.70 ± 0.02	1.22 ± 0.01	0.45 ± 0.01	0.369	0.58	382
A5	4.73 ± 0.1	6.15 ± 0.01	2.1 ± 0.1	0.341	0.52	379
A6	11.60 ± 0.2	17.2 ± 0.1	5.7 ± 0.1	0.331	0.50	377
Samples B—Hydrogels with HEC						
B1	0.04 ± 0.01	0.06 ± 0.01	<0.02	-	-	382
B2	10.73 ± 0.3	11.8 ± 0.1	4.0 ± 0.1	0.339	0.51	385
B3	0.33 ± 0.01	0.41 ± 0.01	<0.10	-	-	380
B4	0.81 ± 0.1	1.03 ± 0.01	0.34 ± 0.01	0.330	0.49	379
B5	5.41 ± 0.01	6.1 ± 0.1	2.0 ± 0.1	0.328	0.49	379
B6	11.28 ± 0.2	13.1 ± 0.1	4.4 ± 0.1	0.336	0.51	375
Samples C—Emulsions						
C1	0.05 ± 0.01	0.07 ± 0.01	<0.02	-	-	380
C2	9.20 ± 0.2	10.5 ± 0.1	3.3 ± 0.1	0.314	0.46	381
C3	0.23 ± 0.01	0.18 ± 0.01	<0.10	-	-	380
C4	0.87 ± 0.1	0.92 ± 0.01	0.35 ± 0.02	0.380	0.61	377
C5	5.39 ± 0.3	6.14 ± 0.01	2.3 ± 0.2	0.375	0.60	375
C6	12.23 ± 0.2	13.4 ± 0.1	4.4 ± 0.1	0.328	0.49	377
Samples D—Emulgels with XG						
D1	0.06 ± 0.01	0.05 ± 0.01	<0.02	-	-	377
D2	7.99 ± 0.2	8.3 ± 0.1	2.7 ± 0.1	0.325	0.48	378
D3	0.25 ± 0.01	0.35 ± 0.01	<0.10	-	-	377
D4	0.64 ± 0.01	0.76 ± 0.01	0.25 ± 0.1	0.329	0.49	378
D5	4.58 ± 0.11	7.3 ± 0.1	2.5 ± 0.1	0.342	0.52	379
D6	8.44 ± 0.31	9.8 ± 0.1	3.1 ± 0.1	0.316	0.46	379
Samples E—Emulgels with HEC						
E1	0.03 ± 0.01	0.04 ± 0.01	<0.02	-	-	378
E2	12.91 ± 0.2	14.3 ± 0.1	4.7 ± 0.1	0.329	0.49	377
E3	0.44 ± 0.01	0.78 ± 0.01	0.23 ± 0.1	0.295	0.42	379
E4	0.85 ± 0.01	0.99 ± 0.01	0.34 ± 0.1	0.343	0.53	375
E5	8.00 ± 0.1	9.4 ± 0.1	3.2 ± 0.1	0.340	0.52	377
E6	13.52 ± 0.4	15.4 ± 0.1	5.5 ± 0.1	0.357	0.56	378

**Table 2:** Qualitative and quantitative compositions (%, (*w*/*w*)) of prepared hydrogel samples with xanthan gum.

INCI Name	Function	A1	A2	A3	A4	A5	A/B6
Xanthan gum	Gel-forming agent	1	1	1	1	1	1
Glycerin	Humectant	10	10	10	10	10	10
Potassium sorbate	Preservative	1	1	1	1	1	1
Ethanol	Solvent	-	8	-	-	4	8
Homosalate	UV filter	-	4	-	-	2	4
Ethylhexyl methoxycinnamate	UV filter	-	4	-	-	2	4
Benzophenone-4	UV filter	-	4	-	-	2	4
NaDES extracts	Cosmetic active ingredient	-	-	12	24	6	12
Sodium hydroxide, 10%	pH regulator	q.s. to adjust pH to 4.5.					
Aqua	Solvent	q.s. ad 100 *	q.s. ad 100 *	q.s. ad 100 *	q.s. ad 100 *	q.s. ad 100 *	q.s. ad 100 *

* quantum satis ad, meaning “a sufficient quantity up to”.

**Table 3:** Qualitative and quantitative compositions (%, (*w*/*w*)) of prepared hydrogel samples with hydroxyethyl cellulose.

INCI Name	Function	B1	B2	B3	B4	B5	B6
Hydroxyethyl cellulose	Gel-forming agent	2	2	2	2	2	2
Glycerin	Humectant	10	10	10	10	10	10
Potassium sorbate	Preservative	1	1	1	1	1	1
Ethanol	Solvent	-	8	-	-	4	8
Homosalate	UV filter	-	4	-	-	2	4
Ethylhexyl methoxycinnamate	UV filter	-	4	-	-	2	4
Benzophenone-4	UV filter	-	4	-	-	2	4
NaDES extracts	Cosmetic active ingredient	-	-	12	24	6	12
Sodium hydroxide, 10%	pH regulator	q.s. to adjust pH to 4.5.					
Aqua	Solvent	q.s. ad 100 *	q.s. ad 100 *	q.s. ad 100 *	q.s. ad 100 *	q.s. ad 100 *	q.s. ad 100 *

* quantum satis ad, meaning “a sufficient quantity up to”.

**Table 4:** Qualitative and quantitative compositions (%, (*w*/*w*)) of prepared emulsion samples.

INCI Name	Function	C1	C2	C3	C4	C5	C6
Olivem 1000^®^ (sorbitan olivate (and) cetearyl olivate)	Emulsifier	5	5	5	5	5	5
Olivem Feel^®^ (cetearyl alcohol (and) cetyl palmitate (and) sorbitan palmitate (and) sorbitan oleate)	Co-emulsifier	1	1	1	1	1	1
Caprylic/capric triglyceride	Emollient	20	20	20	20	20	20
Glycerin	Humectant	8	8	8	8	8	8
Potassium sorbate	Preservative	1	1	1	1	1	1
Homosalate	UV filter	-	4	-	-	2	4
Ethylhexyl methoxycinnamate	UV filter	-	4	-	-	2	4
Benzophenone-4	UV filter	-	4	-	-	2	4
NaDES extracts	Cosmetic active ingredient	-	-	12	24	6	12
Sodium hydroxide, 10%	pH regulator	q.s. to adjust pH to 4.5.					
Aqua	Solvent	q.s. ad 100 *	q.s. ad 100 *	q.s. ad 100 *	q.s. ad 100 *	q.s. ad 100 *	q.s. ad 100 *

* quantum satis ad, meaning “a sufficient quantity up to”.

**Table 5:** Qualitative and quantitative compositions (%, (*w*/*w*)) of prepared emulgel samples with xanthan gum.

INCI Name	Function	D1	D2	D3	D4	D5	D6
Olivem 1000^®^ (sorbitan olivate (and) cetearyl olivate)	Emulsifier	5	5	5	5	5	5
Olivem Feel^®^ (cetearyl alcohol (and) cetyl palmitate (and) sorbitan palmitate (and) sorbitan oleate)	Co-emulsifier	1	1	1	1	1	1
Caprylic/capric triglyceride	Emollient	20	20	20	20	20	20
Glycerin	Humectant	8	8	8	8	8	8
Potassium sorbate	Preservative	1	1	1	1	1	1
Homosalate	UV filter	-	4	-	-	2	4
Ethylhexyl methoxycinnamate	UV filter	-	4	-	-	2	4
Benzophenone-4	UV filter	-	4	-	-	2	4
NaDES extracts	Cosmetic active ingredient	-	-	12	24	6	12
Xanthan gum	Gel-forming agent	0.25	0.25	0.25	0.25	0.25	0.25
Sodium hydroxide, 10%	pH regulator	q.s. to adjust pH to 4.5.					
Aqua	Solvent	q.s. ad 100 *	q.s. ad 100 *	q.s. ad 100 *	q.s. ad 100 *	q.s. ad 100 *	q.s. ad 100 *

* quantum satis ad, meaning “a sufficient quantity up to”.

**Table 6:** Qualitative and quantitative compositions (%, (*w*/*w*)) of prepared emulgel samples with hydroxyethyl cellulose.

INCI Name	Function	E1	E2	E3	E4	E5	E6
Olivem 1000^®^ (sorbitan olivate (and) cetearyl olivate)	Emulsifier	5	5	5	5	5	5
Olivem Feel^®^ (cetearyl alcohol (and) cetyl palmitate (and) sorbitan palmitate (and) sorbitan oleate)	Co-emulsifier	1	1	1	1	1	1
Caprylic/capric triglyceride	Emollient	20	20	20	20	20	20
Glycerin	Humectant	8	8	8	8	8	8
Potassium sorbate	Preservative	1	1	1	1	1	1
Homosalate	UV filter	-	4	-	-	2	4
Ethylhexyl methoxycinnamate	UV filter	-	4	-	-	2	4
Benzophenone-4	UV filter	-	4	-	-	2	4
NaDES extracts	Cosmetic active ingredient	-	-	12	24	6	12
Hydroxyethyl cellulose	Gel-forming agent	0.5	0.5	0.5	0.5	0.5	0.5
Sodium hydroxide, 10%	pH regulator	q.s. to adjust pH to 4.5.					
Aqua	Solvent	q.s. ad 100 *	q.s. ad 100 *	q.s. ad 100 *	q.s. ad 100 *	q.s. ad 100 *	q.s. ad 100 *

* quantum satis ad, meaning “a sufficient quantity up to”.

## Data Availability

The original contributions presented in this study are included in the article. Further inquiries can be directed to the corresponding author.
